# Arterial switch operation: progress in approaching a zero mortality rate

**DOI:** 10.1186/1749-8090-8-S1-O307

**Published:** 2013-09-11

**Authors:** K Drozdovski, A Bashkevitch, Y Chesnov, V Dedovich, Y Linnik, E Karalkova, I Turchinova, L Yauhrafava, W Novick

**Affiliations:** 1 Pediatric Surgery, National Children's Cardiac Surgical Center, Minsk, Belarus; 2 Pediatric Cardiac Surgery, National Children's Cardiac Surgical Center, Minsk, Belarus; 3 Department of Surgery, University of Tennessee Health Sciences Center and International Children's Heart Foundation, Memphis, TN, USA

## Background

The learning curve for the arterial switch operation (ASO) has been reported to be steep. We reviewed our entire experience in the ASO from 1996 to determine the change in mortality over time and determine what factors, if any, resulted in our current annual mortality of 0%.

## Methods

The database of our institution was reviewed for all children undergoing the arterial switch procedure. All ASO’s were included; ASO/IVS, ASO/VSD, ASO/arch repair, ASO/VSD/arch repair. Multivariate analysis of those factors which might have an influence on mortality was performed.

## Results

A total of 163 ASO procedures of all types were identified. The diagnoses were; TGA/IVA 88, TGA/VSD 53, TGA/arch 5, TGA/VSD/arch 4, Taussig Bing 10, Taussig Bing/arch 9, for a total of 169, ten patients underwent a staged repair in preparation for ASO of which 4 died. One died before intervention, one did not undergo ASO operation at our institution. Total mortality for all ASO procedures was 23% (38/163). Mortality over time decreased from 100% in 1996 to 0% in 54 consecutive patients between June 2009 and December 2012. A linear relationship in mortality was noted over time, with a correlation coefficient of 0.88. Analysis of other factors including; coronary anatomy, type of ASO, cross-clamp time did not reveal significant differences. The ASO/VSD operation was initially a risk factor with an overall mortality of 28% (15/53), however this disappeared over time.

## Conclusion

A significant learning curve is required to achieve low mortality in the ASO. Early failure should not discourage pursuit of improved results.

